# A tumor microenvironment preoperative nomogram for prediction of lymph node metastasis in bladder cancer

**DOI:** 10.3389/fonc.2022.1099965

**Published:** 2022-12-15

**Authors:** Zhenghao Chen, Chuan Qin, Gang Wang, Donghao Shang, Ye Tian, Lushun Yuan, Rui Cao

**Affiliations:** ^1^ Department of Urology, Beijing Friendship Hospital, Capital Medical University, Beijing, China; ^2^ Department of Biological Repositories, Zhongnan Hospital of Wuhan University, Wuhan, China; ^3^ Department of Internal Medicine, Division of Nephrology, Leiden University Medical Center, Leiden, Netherlands

**Keywords:** tumor microenvironment, bladder cancer (BLCA), lymph node metastasis (LNM), prognosis, preoperative nomogram

## Abstract

**Background:**

Growing evidence suggests that tumor metastasis necessitates multi-step microenvironmental regulation. Lymph node metastasis (LNM) influences both pre- and post-operative bladder cancer (BLCA) treatment strategies. Given that current LNM diagnosis methods are still insufficient, we intend to investigate the microenvironmental changes in BLCA with and without LNM and develop a prediction model to confirm LNM status.

**Method:**

"Estimation of Stromal and Immune cells in Malignant Tumors using Expression data" (ESTIMATE) algorithm was used to characterize the tumor microenvironment pattern of TCGA-BLCA cohort, and dimension reduction, feature selection, and StrLNM signature construction were accomplished using least absolute shrinkage and selection operator (LASSO) regression. StrLNM signature was combined with the genomic mutation to establish an LNM nomogram by using multivariable logistic regression. The performance of the nomogram was evaluated in terms of calibration, discrimination, and clinical utility. The testing set from the TCGA-BLCA cohort was used for internal validation. Moreover, three independent cohorts were used for external validation, and BLCA patients from our cohort were also used for further validation.

**Results:**

The StrLNM signature, consisting of 22 selected features, could accurately predict LNM status in the TCGA-BLCA cohort and several independent cohorts. The nomogram performed well in discriminating LNM status, with the area under curve (AUC) of 75.1% and 65.4% in training and testing datasets from the TCGA-BLCA cohort. Furthermore, the StrLNM nomogram demonstrated good calibration with p >0.05 in the Hosmer-Lemeshow goodness of fit test. Decision curve analysis (DCA) revealed that the StrLNM nomogram had a high potential for clinical utility. Additionally, 14 of 22 stably expressed genes were identified by survival analysis and confirmed by qPCR in BLCA patient samples in our cohort.

**Conclusion:**

In summary, we developed a nomogram that included an StrLNM signature and facilitated the preoperative prediction of LNM status in BLCA patients.

## 1 Introduction

Bladder cancer (BLCA) is one of the most common malignant tumor of the urinary tract (1). The incidence and mortality of BLCA are increasing due to factors such as increasing age, environmental pollution, smoking, and other factors (2). Lymph node metastasis (LNM) is a major means of metastasis of BLCA and several theories have been proposed to explain the mechanism of LNM in BLCA, including lymphangiogenesis, epithelial-mesenchymal transition (EMT), cell invasion, and tumor microenvironment (3). Previous studies have shown that LNM status is an independent unfavorable prognostic factor in BLCA patients (4–6), and the five-year overall survival (OS) is considerably lower in patients with LNM than in patients without LNM (28% vs. 54%) (7, 8).

Given that there is such a remarkable difference in prognosis between LN-positive and LN-negative patients, In clinical practice, preoperative assessment of LN status is important. Current diagnostic procedures based on traditional radiology, including computerized tomography (CT) and magnetic resonance imaging (MRI).Though the specificity of diagnosis LNM by these method is (91.9% for CT and 89%–98% for MRI), but the sensitivity is relatively low (36.9% for CT and 76–83% for MRI) to preoperatively predict the lymphatic metastasis of BLCA patients (9, 10). Various groups have attempted to develop a nomogram based on CT as well as other radiomics to predict the status of LNM in BLCA patients. For example, Wu et al. established a nomogram based on radiomics features extracted from arterial-phase CT images. However, this nomogram lacked external validation and was constructed without the use of genetic markers, thus its clinical significance needed to be confirmed further (11). As for positron emission tomography (PET), although PET/CT technology plays an increasingly important role in the preoperative evaluation of clinical patients, both F18-PET/CT (12) and 11C-choline PET/CT (13) were not doing well in the sensitivity of lymph node assessment. What’s more, considering the gap between economic development and medical resources in different countries and regions, it is unrealistic to routinely use PET/CT as a preoperative auxiliary examination for BLCA patients. Thus, it is necessary to establish a new noninvasive and effective preoperative assessment to predict LNM in patients.

Furthermore, the interaction between BLCA cells and other cells in the TME contributes to lymph node metastasis. Recognizing that the TME is characterized in cancer development has changed our understanding of cancer development from a cancer cell-centric perspective to tumor growth and metastasis supporting advanced tumor ecosystems (14). Rather than working alone, tumor cells build particular TME through intimate interactions with the extracellular matrix (ECM) as well as stromal cells (15). Lymphatic endothelial cells could express CCL21 and attract CCR7-expressing tumor cells to lymphatic vessels and present antigens (16). Numerous immune and non-immune cells in the TME infrastructure, along with the factors they secrete, contribute to chronic inflammation, immunosuppression, and the gastrointestinal milieu (17).

With the rapid development of next-generation sequencing technology and the comprehensive study of human transcriptome and gene changes, it has become possible to discover the differences of BLCA in LNM from the genomic level. In recent decades, we have seen a shift from a single analysis of multiple biomarkers to the combinatorial analysis of a panel of biomarkers to construct markers, which is regarded as an useful and effectivetool for clinical management methods (18). Our team previously built a nomogram combining an EMT-LNM signature and somatic genetic mutations for prediction of LNM in BLCA which was pretty useful for clinical practice (19). And in the current study, we focused on TME patterns and tried to establish a nomogram which was centered on an StrLNM signature for preoperative prediction of LNM status in BLCA patients.

## 2 Material and methods

### 2.1 Data collection and processing

The TCGA-BLCA dataset was obtained from the TCGA Genomic Data Commons (GDC) (https://portal.gdc.cancer.gov/) and was used as the training and internal validation cohort (**Table S3, S4**). Since multiple ENSEMBL IDs mapped to a single gene symbol, the highest expressed ENSEMBL ID was used. TCGA-BLCA fragments per kilobase million (FPKM) values were downloaded from the TCGA GDC and transformed into transcripts per kilobase million (TPM) (20). Details of the clinicopathological features of each dataset were obtained in our previous study (21). We included a total of 256 samples in our study including 128 lymph node metastases (LN+) and 228 non-lymph node metastases (LN-)as the total TCGA-BLCA cohort. Three distinct BLCA cohorts, GSE13507 cohort (22), GSE31684 cohort (23), and GSE106534 cohort (24), were acquired for external validation. “Affyy” R packages was used in R for the log2 transformation, background correction, annotation, and quantile normalization to process the raw data (25).

### 2.2 Identification of differentially expressed genes (DEGs)

The differential expression genes of LN+ and LN- samples was evaluated, as well as high Stromal score samples and low Stromal score samples, in the TCGA-BLCA cohort by R package “DESeq2”. The significant cutoff for defining DEGs was set as |log2Fold Change (FC)| >1.0 and false discovery rate (FDR) < 0.05. Among all the DEGs both upregulated or downregulated genes in LN+/LN- groups and high/low Stromal groups were defined as the final DEGs.

### 2.3 Generation of StrLNM signature

Primary predictive features was built by the Least absolute shrinkage and selection operator (LASSO) logistic regression analysis, and screened final DEGs was used to build an StrLNM signature by the R package “glmnet” (26). The best value of the penalty parameter λ was determined through 10-fold cross-validation error for dimension reduction to reduce noise or redundant genes. The risk value for each sample’s StrLNM signature was calculated from a linear combination of selected features, weighted with an appropriate coefficient. The equation of risk value= $\sum_{i=1}^{n}$ (coefi × Expri), where Expri is the relative expression of the genes in the signature for patients i and coefi was the relevant coefficients of the genes. And we further used U test to explore the correlation of StrLNM and LNM in testing dataset.

### 2.4 Development of an individualized prediction model

Univariable and multivariable logistic regression analysis were further used to development the model with candidate features including the StrLNM signature and C3orf70. StrLNM nomograms were generated using the “rms”, “nomogrammex”, and “regplot” R packages as a quantitative tool for clinicians to predict individual LNM probabilities.

### 2.5 Validation of StrLNM signature and nomogram

After the StrLNM signature was established, internal validation was performed on the TCGA-BLCA cohort test dataset according to the risk assessment formula specified in the training dataset. The nomogram was then tested for predictive accuracy and stability using receiver operating characteristic (ROC) curves and calibration curves by using R packages “pROC” and “rms”. Moreover, three independent GEO cohorts (GSE13507, GSE31684, and GSE106534) were used for the external validation. A supervised hierarchical clustering method was used to explore clusters with k = 2 based on 1-Pearson’s correlation distance.

### Tissue specimens

As previously described, a total of 55 BLCA specimens were randomly recruited and paired adjacent normal tissue samples were obtained at the Beijing Friendship Hospital, Capital Medical University (Beijing, China) between January 2021 and March 2022. The clinical characteristic of our specimens was listed in **Table 1**. And there was no attrition during the study.

**Table 1:** Characteristic of patients.

Characteristic of patients		
Gender		
	Male	48
	Female	7
Age		67.8 ± 10.7
T		
	T1	9
	T2	17
	T3	22
	T4	7
N		
	N0	39
	N+	16
Grade		
	High Grade	42
	Low Grade	13

The inclusion criteria for our specimens were patients with a clinical and histological diagnosis of BLCA.And the exclusion criteria were patients who had received any chemotherapy, neoadjuvant chemotherapy, radiotherapy and immunotherapy. Patients with severe urinary tract infection, severe renal failure, serious underlying diseases of other systems, or who had participated in other studies were also excluded. This study was approved by the Ethics Committee of the Affiliated Friendship Hospital of Capital Medical University (NO.2021-P2-159). The patients provided their written informed consent to participate in this study.

### 2.6 RNA extraction, reverse transcription, and quantitative real-time PCR (qRT-PCR)

During the whole process of RNA extraction and PCR, operators follow the principle of blind method. Total RNA of clinical samples was extracted by the RNeasy plus mini kits (74136, Qiagen, Germany) Subsequently, NanoDrop instrument (NP80, Implen, Germany) was used to exam the quality of the extracted RNA. Then, the RNA was used as a template for cDNA synthesis using the ReverTra Ace qPCR RT Kit (FSQ-201, Toyobo, Japan). Finally, forward and reverse primers of our key genes and iQTM SYBR^®^ Green Supermix (1708880, Bio-Rad, US) were mixed, and performed the qRT-PCR. The primer sequences were listed in **Table S1**. The relative expression level of the targeted genes was normalized to GAPDH as described by Schmittgen TD et al. 2-△△CT=[(CT gene of interest - CT internal control) sample A - (CT gene of interest - CT internal control) sample B] (27).

### 2.7 Statistical analyses

The statistical significance of data between two groups was tested by Student’s t-test, Mann-Whitney U test, Fisher’s exact and χ2 tests according to the data type. We used R package “survival” and “survminer” to generate Kaplan-Meier curves and Cox regression for survival analysis (28). The significant difference between survival curves belonging to different defined groups were determined with the log-rank (Mantel-Cox) test. And we used R packages “rms,” “nomogramEx,” and “regplot” to build nomogram and calibration curves (21). Decision curve analysis (DCA) was performed to determine whether our established nomogram was of clinical usefulness according to Iasonos et al.’s suggestion (29). The package “pROC” in R was used to plot and visualize ROC curves. All statistical analyses were performed with R software 3.5.3. Statistical cutoff point was set at p < 0.05.

## 3 Results

### 3.1 Characterization of patients in BLCA

**Figure 1:**
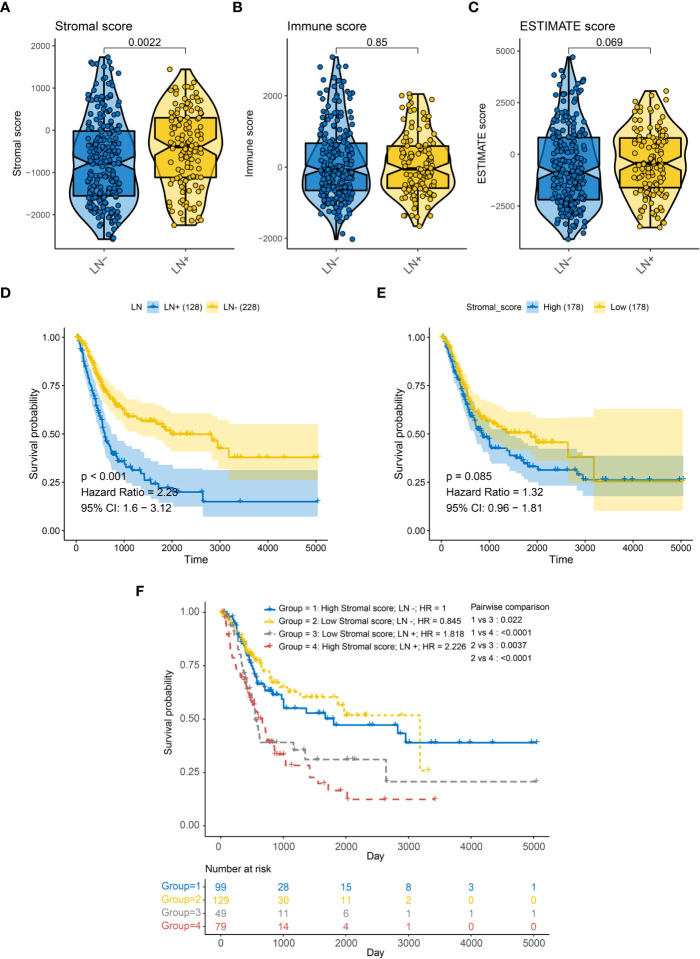
Association between LN metastasis status and TME related score of BLCA patients in the TCGA-BLCA cohort for overall survival (OS). **(A)** Correlation between LN metastasis and Stromal score. **(B)** Correlation between LN metastasis and immune score. **(C)** Correlation between LN metastasis and ESTIMATE score. **(D)** Kaplan-Meier survival curves of LN metastasis **(E)** Kaplan-Meier survival curves of Stromal score **(F)** Kaplan-Meier survival curves show the difference in prognosis advantage among four groups stratified by LN metastasis and Stromal score.

We first assessed Stromal score, Immune score, and Estimated score in 356 patients with or without LNM in the TCGA-BLCA cohort (**Table S2**). Stromal score were significantly higher in patients with LNM (**Figure 1A**, p = 0.0022); However, Immune score and Estimated score did not differ significantly between these patients (**Figures 1B, C**). And we further examined overall survival in patients with or without LNM, and it is clear that LNM is an unfavorable prognostic factor (**Figure 1D**). Also, patients with a higher Stromal score had a potential for poorer overall survival (OS) (**Figure 1E**). Therefore, we divided all patients into four groups according to the Stromal score and the LNM status. Interestingly, regardless of the level of Stromal score, Kaplan-Meier survival curves showed significantly improved survival in LN- patients, and patients without LNM and low Stromal score level had a better prognosis than other groups (log-rank test, p < 0.05, **Figure 1F**). And the Recurrence free survival (RFS) curve was similar to OS and was shown in **Supplementary Figure S1**.

### 3.2 DEGs between LN+/- and high/low Stromal score patients

**Figure 2:**
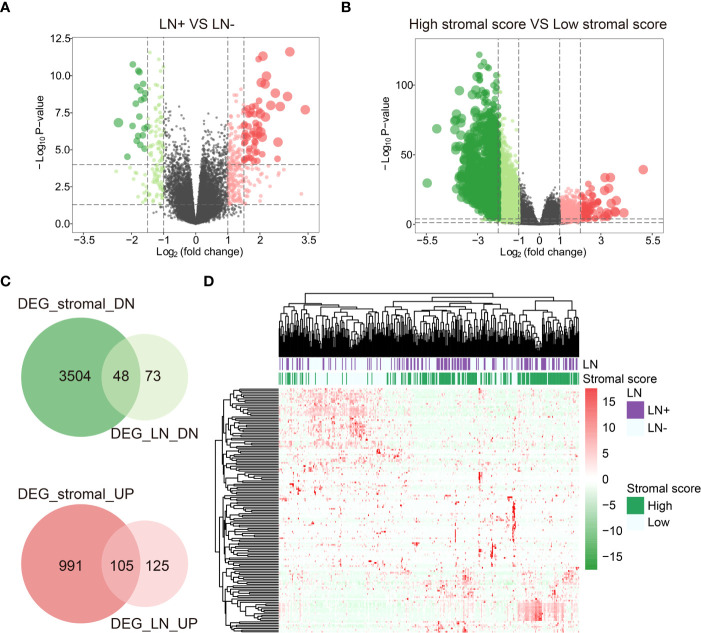
DEGs between LN+/- and high/low Stromal score patients. **(A)** DEGs between LN+ and LN-. **(B)** DEGs between high/low Stromal score patients. **(C)** DEGs both involved in Stromal score and LN. **(D)** Heatmap of DEGs both involved in Stromal score and LN.

When comparing LN+ and LN-, we screened 121 down-regulated DEGs (DEG_LN_DN) and 230 up-regulated DEGs (DEG_LN_UP) by the package “DESeq2” in R (**Figure 2A**). In addition, we identified 3552 down-regulated differentially expressed genes (DEG_stromal_DN) and 1096 up-regulated differentially expressed genes (DEG_stromal_UP) between patients with high and low Stromal score (**Figure 2B**). Among all these DEGs there were 48 DEGs both involved in DEG_stromal_DN and DEG_LN_DN, and 105 DEGs participated in both DEG_stromal_UP and DEG_LN_UP (**Figure 2C**). Therefore, we defined these 153 genes as the most important genes among LN+/LN- patients. The heatmap showed the expression levels of 153 DEGs in patients was characterized by LN+/LN- and Stromal score (**Figure 2D**).

### 3.3 Establishment of StrLNM-Signature

**Figure 3:**
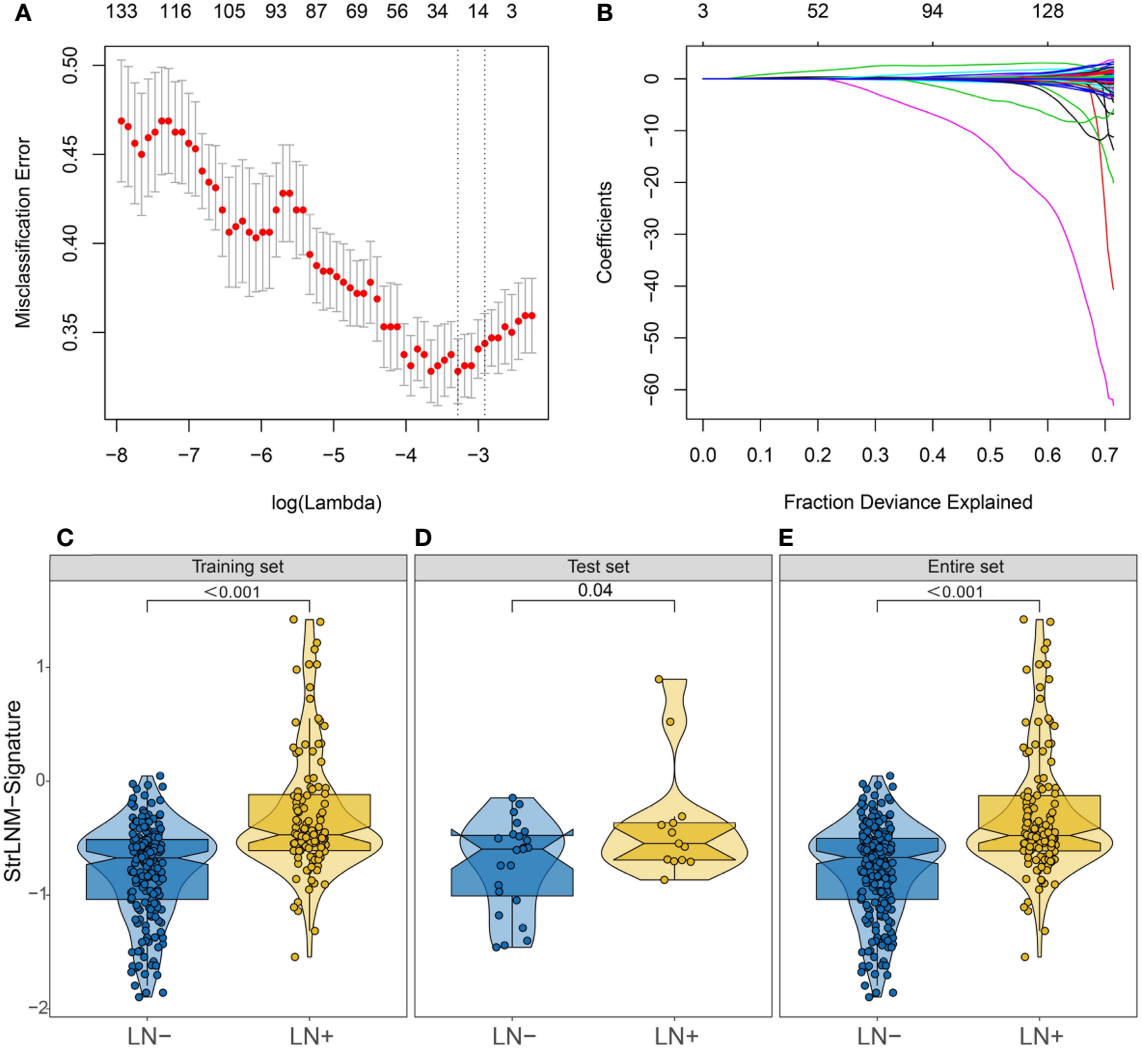
Feature selection using LASSO logistic regression model. **(A)** Tuning parameter (λ) selection 10-fold cross-validation error curve. The misclassification error was plotted vs. log (λ). The dotted vertical lines were drawn at the optimal values by the minimum criteria and the 1-SE criteria. **(B)** LASSO coefficient profiles of the 19 candidate EMT-related genes. A coefficient profile plot was produced against the log (λ) sequence. A vertical line was drawn at the value selected by 10-fold cross-validation, where the optimal λ resulted in 17 nonzero coefficients. **(C–E)** Difference in the StrLNM signature risk score between LN+ and LN- tumors in the training dataset **(C)**, testing dataset **(D)** and entire set **(E)** of the TCGA-BLCA cohort.

We further performed LASSO regression analysis on 153 DEGs and constructed a signature (StrLNM signature) to distinguish LNM status in the TCGA-BLCA cohort training dataset (**Figures 3A, B**). The coefficient of each feature in the StrLNM signature was shown in **Table S5**. Furthermore, the StrLNM signature was significantly higher in LN+ tumors than in LN tumors in both training(p <0.001) and test datasets(p = 0.04) of the TCGA-BLCA cohort, and the entire dataset also matched well with the same results (**Figures 3C–E**).

### 3.4 Supervised hierarchical clustering according to StrLNM signature

**Figure 4:**
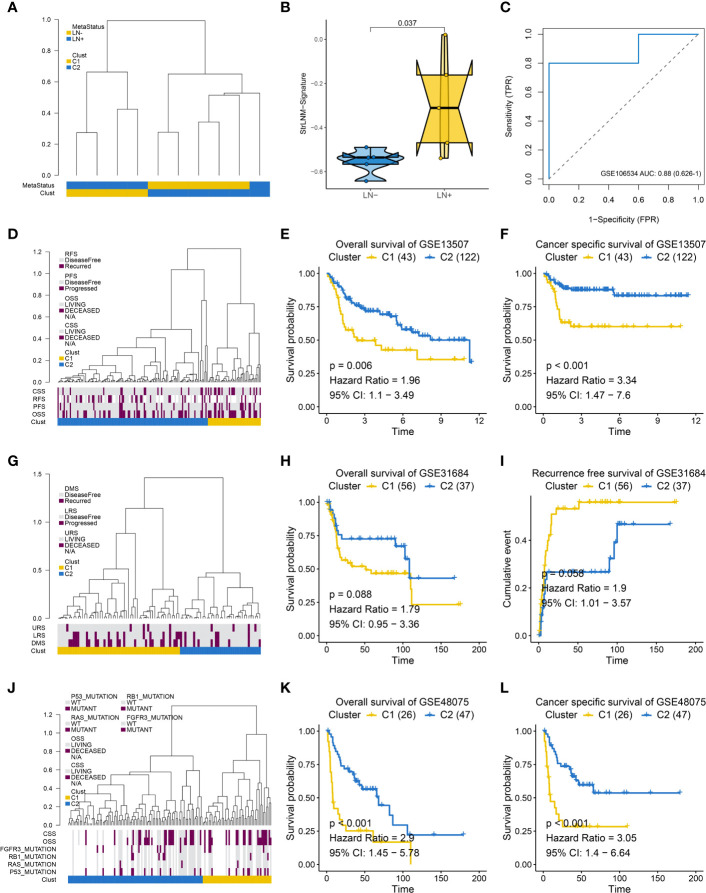
Supervised hierarchical clustering for EMT-LN signature. **(A)** Dendrogram showed that two clusters created by supervised hierarchical clustering could significantly distinguish LN metastasis status in the GSE106534 cohort. **(B)** different StrLNM signature score in LN+ and LN- patients. **(C)** ROC curves of StrLNM signature score in the GSE106534 cohort. **(D)** Dendrogram showed that two clusters created by supervised hierarchical clustering were strikingly associated with OS, RFS, and CSS in the GSE13507 cohort. **(E, F)** Kaplan-Meier survival curves showed the difference in OS (log-rank test, p =0.006, **E**) and CSS (log-rank test, p < 0.001, **F**) between LN+ and LN- tumors in the GSE13507 cohort. **(G)** Dendrogram showed that two clusters created by supervised hierarchical clustering were strikingly associated with disease metastasis survival (DMS), lymph node recurrence survival (LRS), and urinary tract recurrence survival (URS) in the GSE31684 cohort. **(H, I)** Kaplan-Meier survival curves showed the difference in OS (log-rank test, p =0.088, **H**) and RFS (log-rank test, p =0.056, **I**) between LN+ and LN- tumors in the GSE31684 cohort. **(J)** Dendrogram showed that two clusters created by supervised hierarchical clustering were strikingly associated with P53, RAS, RB1 and FGFR3 mutation as well as OS and CSS in the GSE48075 cohort. **(K, L)** Kaplan-Meier survival curves showed the difference in OS (log-rank test, p < 0.001, **K**) and CSS (log-rank test, p < 0.001, **L**) between LN+ and LN- tumors in the GSE48075 cohort.

In addition, we performed hierarchical clustering to see whether StrLNM signature could discriminate LNM in the other three external cohorts. We found cluster C1 was mainly concentrated in LN+ tumors, whereas the cluster C2 gathered in LN- patients in the GSE106534 cohort (**Figure 4A**). When we further explored the StrLNM signature score in LN+ and LN- patients, it was obvious that LN+ patients had a higher StrLNM signature score (**Figure 4B**). The area under curve (AUC) of the StrLNM signature score in the GSE106534 cohort was 0.88 (**Figure 4C**). We then further verified the StrLNM cluster in the other three datasets (GSE13507, GSE31684 and GSE48075) and confirmed that cluster C1 which almost all consisted of LN+ had a significantly worse OS and cancer-specific survival (CSS) than cluster C2 patients in GSE13507(**Figures 4D–F**), in GSE31684 (**Figures 4G–I**), and in GSE48075 (**Figures 4J–L**). These data suggested that stromal-rich BLCA patients were prone to have LNM and worse outcomes.

### 3.5 Individualized prognostic nomogram: Development and validation

Logistic regression analysis was used to determine the predictive characteristics of preoperative lymph node metastasis. Considering that the pathological staging and detailed classification of TNM are more obtained by postoperative pathological examination than preoperative evaluation, in this study we included the StrLNM signature and mutation of C3orf70, which were proved to be obviously highly mutated in LN+ tumors when compared with LN- tumors (**Table S6**) (19), as the candidate features to build the preoperative nomogram.

**Figure 5:**
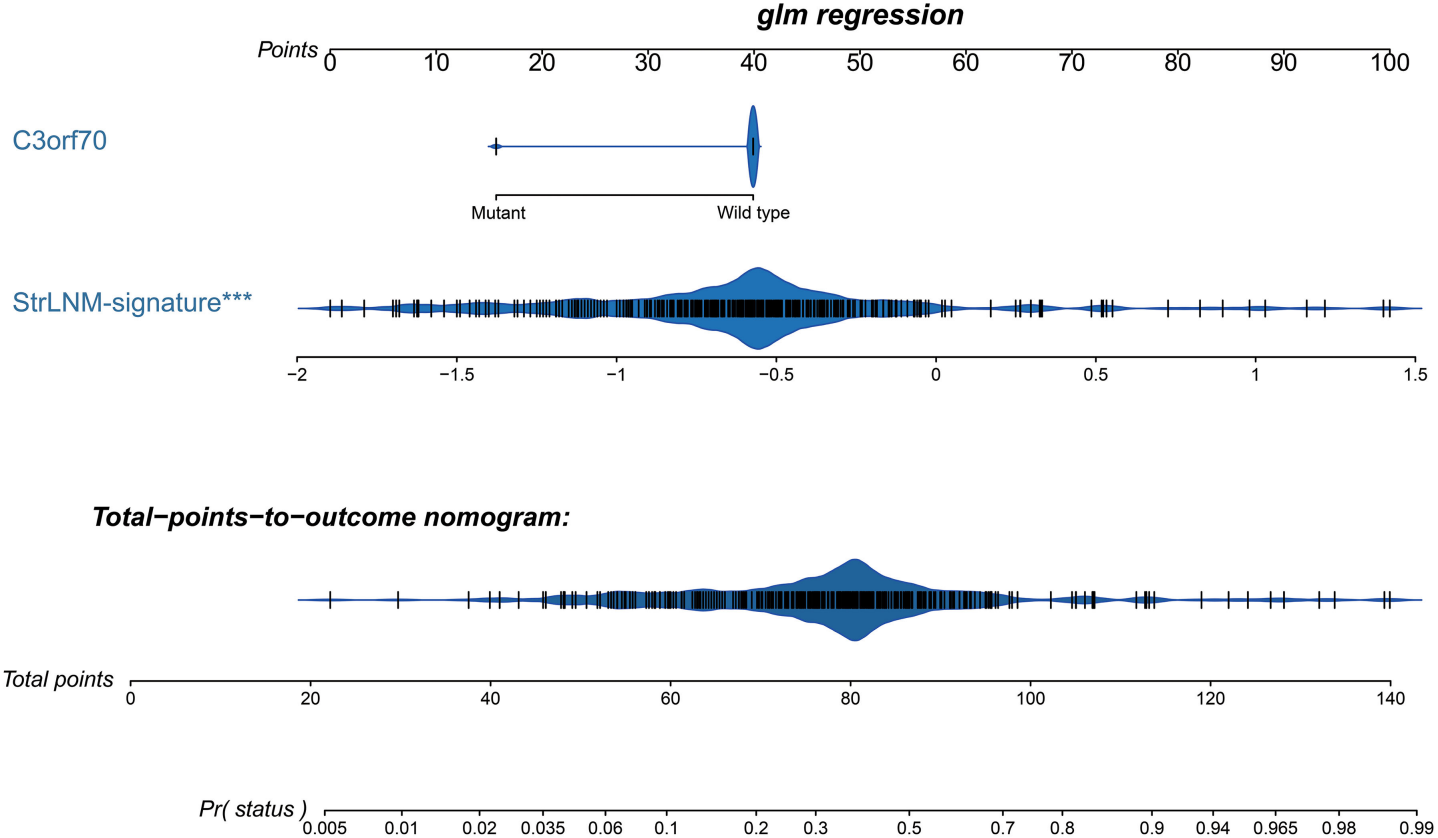
Development of preoperative StrLNM nomogram. By incorporating the StrLNM signature and genomic mutation of C3orf70, the StrLNM nomogram was built in the training dataset of the TCGA-BLCA cohort.

**Figure 6:**
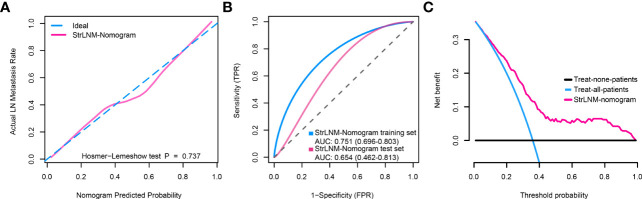
Clinical utility of the StrLNM nomogram. **(A)** Calibration curve of the StrLNM nomogram in the training dataset of the TCGA-BLCA cohort, which depicts the calibration of the fitted model between the predicted risk of LN metastasis and actual LN metastasis rate. The x-axis represents the predicted LN metastasis risk, and the y-axis represents the actual LN metastasis rate. The pink solid line represents the performance of the StrLNM nomogram, of which a closer fit to the diagonal dotted blue line represents an ideal prediction. The difference in the two models was measured with the Hosmer-Lemeshow test. **(B)** ROC curves showed the prediction accuracy of the StrLNM signature in prediction of the LN metastasis in training and testing datasets of the TCGA-BLCA cohort. **(C)** Decision curve analysis (DCA) for the StrLNM nomogram. The y-axis measures the net benefit. The pink line represents the StrLNM nomogram, the blue line represents the assumption that all patients have LN metastases, and the black line on the bottom represents the assumption that no patients have LN metastases.

Logistic regression found the StrLNM signature and C3orf70 mutation were statistically significant (p < 0.05) (**Table S6**). Therefore, we synthesize these features to form the StrLNM nomogram (**Figure 5**). The scores for each prognostic parameter for each patient were summed to obtain a complete score according to the nomogram. The higher the complete score, the more chance of LNM. The nomogram calibration curve then shows that the model performs similarly to the ideal model in the training set (p = 0.737) (**Figure 6A**). ROC curves (**Figure 6B**) showed that the StrLNM nomogram could effectively predict the LNM, with high AUCs in both training dataset (AUC: 0.751 [0.696−0.803]) and testing dataset (AUC: 0.654 [0.462−0.813)). Then, DCA curve showed that the net benefit of the StrLNM nomogram was higher than that of the “complete treatment” or “no treatment” strategy, suggesting that the StrLNM nomogram had a higher potential clinical application value (**Figure 6C**).

### 3.6 Expression level of StrLNM candidate genes in BLCA patient samples

**Figure 7:**
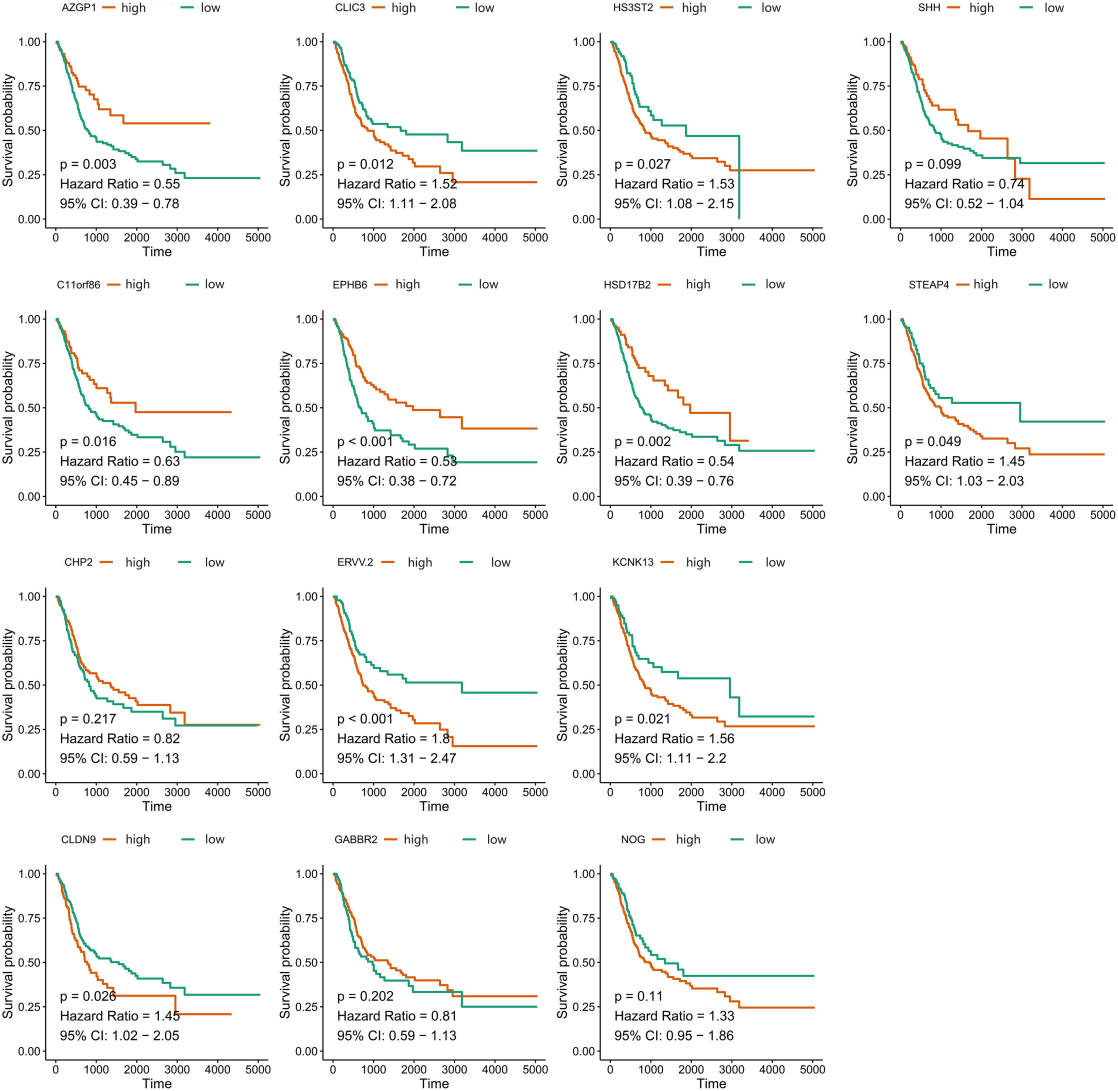
Overall survival of 14 stably-expressed genes. Kaplan-Meier survival curves showed the different correlation between OS and expression level of candidate genes.

A total of 55 BLCA specimens and paired adjacent normal tissue samples, including 16 LN+ samples and 39 LN- samples were obtained. We validated the expression of 14 stably expressed candidate genes (over 50% samples detected in the TCGA-BLCA cohort) in BLCA patients’ samples by qPCR and examined the association between these genes and survival in the TCGA-BLCA cohort (**Figure 7**). AZGP1, C11orf86, CLDN9, CLIC3, EPHB6, ERVV.2, HS3ST2, HSD17B2, KCNK13, and STEAP4 showed a significant correlation with survival while the p-value of SHH was also close to 0.05 (**Table S7**). We then further explored the expression of these genes between LN+ and LN- BLCA patients, which showed that the expression level of AZGP1, C11orf86, HSD17B2, and SHH in LN+ patients were significantly lower than in LN- patients, meanwhile, CLIC3 and KCNK13 were both highly expressed in LN+ patients.

## 4 Discussion

In the present study, we focused on stromal cells of TME and tried to establish a nomogram combining an StrLNM signature for preoperatively predict LNM and confirmed that AZGP1, C11orf86, CLIC3, HSD17B2, KCNK13, and SHH were potentially predictive biomarkers in BLCA. These results indicate that stromal infiltration involvement in LNM and the potential clinical utility of our StrLNM signature.

Cancer research has recently shifted from focusing solely on tumor cells to comprehensively understanding the surroundings of core tumor cells, known as the TME (30–32). Tumorigenesis may be a complex and dynamic process with three stages: initiation, progression, and metastasis, and the physiological state of the TME was found to be closely related to the various stages of tumorigenesis. TME immune cell infiltration *in situ* has been identified as a piece of important and valuable information for predicting the prognosis and immunotherapy of various cancers in clinical studies of ICIs (33–36). Multiple studies have demonstrated the important role of TME in determining prognosis and LNM in BLCA (24, 33, 37). These studies demonstrated the enormous potential of TME in BLCA prognosis and preoperative evaluation, which was consistent with our findings that higher stromal infiltration was found in LN+ BLCA and associated with worse outcomes.

Multiple studies have found that lymph node dissection can improve the prognosis of BLCA patients at various stages of tumor growth, implying the importance of a more accurate LNM assessment strategy (38, 39). And in our study, to reduce noise and redundant features, we used the LASSO algorithm to combine all features into a single feature, the marker panel, rather than a single predictor selection based on the strength of univariate regression analysis. The StrLNM signature identified in the current study was distributed significantly differently in LN+ and LN- tumors in the TCGA-BLCA training, test cohorts as well as GEO cohorts, indicating our StrLNM signature could be considered as a non-invasive factor for our preoperative prediction of lymph node metastasis. The nomogram and DCA curves were also used to confirm the predictive value of our StrLNM signature. The nomogram is a quantification tool, and people get an overall score by adding points to each nomogram feature, which calculated the contribution of features according to special algorithms. And in this study, our nomogram was pretty accurate in diagnosing lymph node metastases in the TCGA-BLCA training group (75.1%) and test group (65.4%) compared with 36.9% sensitivity for CT (9). Therefore, our StrLNM nomogram could effectively predict the LNM of patients before operation, and provide an effective auxiliary role for follow-up treatment. Furthermore, DCA curves revealed that our nomogram-based decision-making was superior to all patients with and without treatment, with a higher threshold probability and better clinical outcomes. All of these findings suggested that our developed nomogram had a high potential for clinical application.

**Figure 8:**
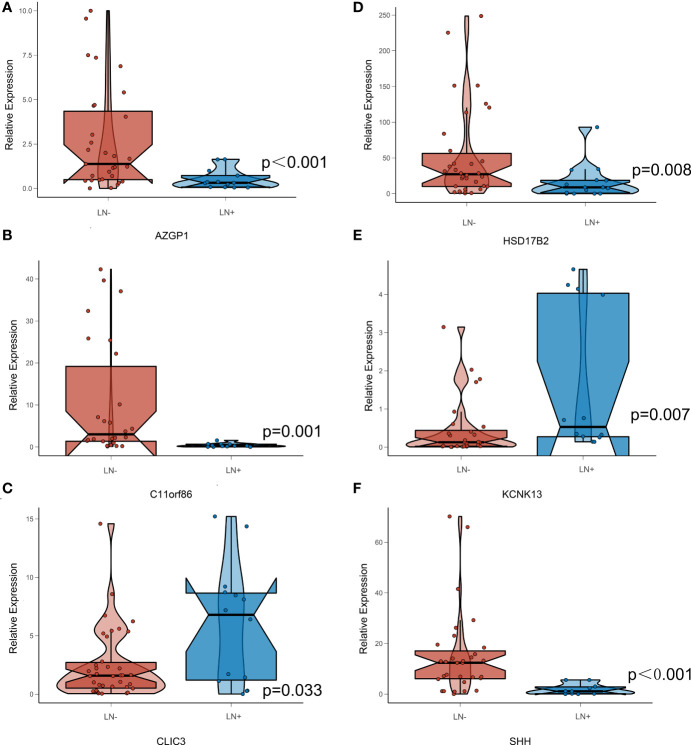
Expression Level of StrLNM candidate genes in BLCA patients’ sample. **(A)** Expression level of AZGP1 between LN+ and LN- samples. **(B)** Expression level of C11orf86 between LN+ and LN- samples. **(C)** Expression level of CLIC3 between LN+ and LN- samples. **(D)** Expression level of HSD17B2 between LN+ and LN- samples. **(E)** Expression level of KCNK13 between LN+ and LN- samples. **(F)** Expression level of SHH between LN+ and LN- samples.

Moreover, we found the expression levels of AZGP1, C11orf86, HSD17B2, SHH, CLIC3, and KCNK13 were significantly different between our own BLCA patients’ tumor and para-carcinoma tissues using qPCR, and the expression level of AZGP1, C11orf86, HSD17B2 and SHH in LN+ patients were significantly lower which indicates they all had a positive effect on patients’ prognosis. And in our StrLNM-Signature system, these genes contributed negatively to the StrLNM score. Since we had proved that LN+ patients had a higher StrLNM signature score, we believed AZGP1, C11orf86, HSD17B2 as well as SHH were potentially important biomarker candidates that were significantly associated with LNM and would contribute to the prediction of BLCA patients. On other hand, CLIC3, and KCNK13 involved the same result from a different angle. They were both highly expressed in LN+ patients indicating they both had a negative effect on prognosis while these genes contributed positively to our StrLNM score. As a matter of fact, Wang et al. had already reported the correlation between highly expressed HSD17B2 and better prognosis in bladder (40). And though there was little research in BLCA, there were a series of researches indicating the role of AZGP1 in various cancers. Kong B et al. found Zinc α2-glycoprotein (AZGP1, ZAG) acts as a tumor suppressor in pancreatic ductal adenocarcinoma, and its expression is lost due to histone deacetylation (41). The sonic hedgehog (SHH) signaling pathway, an evolutionarily conserved molecular cascade that is primarily involved in the development of the fetal central nervous system, was a well-documented trail along the bladder. And it was reported to be correlated with tumorigenesis, EMT, and BLCA stemness (42–44). Interestingly, as a canonical potassium channel, KCNK13 was rarely described in tumors. We all know that ion channels such as transient receptor potential (TRP) channels have been shown to play an important role in tumorigenesis and invasion in pan-cancer, and KCNK13 channels, another classic cation channels, deserve further study. These six genes depicted in **Figure 8** showed a significant correlation in both LNM assessment and tumor/para-carcinoma tissue expression, indicating that these genes play a more important role in LNM and tumorigenesis and warrant more research.

As for clinical use of our StrLNM signature, cystoscopy is necessary for the diagnosis of bladder cancer according to the guideline of European association (45). Also, histological evaluation of resected tissue was also recommended for ultimately diagnosis. Thus, in the process of preoperative cystoscopy, the patient’s BLCA tissue can be easily obtained, and the corresponding simple key gene expression level detection can be carried out, and then the score can be directly obtained for clinical reference.

In this study, we first introduced TME into our StrLNM signature, combined a large external dataset to validate the results, and validate our key genes using patient materials. Nevertheless, there was also a limitation in our study. Our research has not been fully integrated with techniques such as radiomics, and practical clinical applications are still a long way off. And at this stage, it is not available for us to carry out *in vitro* and *in vivo* experiments to further verify the functions of our key genes.

## 5 Conclusion

In conclusion, we created an StrLNM nomogram with StrLNM signature, which is an easily accessible and convenient tool to help preoperatively predict LN metastasis in BLCA patients.

## Data availability statement

The datasets presented in this study can be found in online repositories. The names of the repository/repositories and accession number(s) can be found in the article/**Supplementary Material**.

## Ethics statement

This study was approved by the Ethics Committee of the Affiliated Friendship Hospital of Capital Medical University.(NO.2021-P2-159). The patients/participants provided their written informed consent to participate in this study.

## Author contributions

LY and RC made substantial contributions to the conception and design of the research. ZC, CQ, and GW integrated and analyzed the data. ZC wrote the paper. DS, YT, LY, and RC edited the manuscript and provided critical comments. All authors read and approved the final manuscript.
