# Supplementary material for: A tumor microenvironment preoperative nomogram for prediction of lymph node metastasis in bladder cancer
Source: Front Oncol. 2022 Dec 15;12:1099965. doi: 10.3389/fonc.2022.1099965 (PMC9798213; doi:10.3389/fonc.2022.1099965)
Supplement: Supplementary file 1 [file DataSheet_1.zip › description of supplementary material.docx]

Figure S1. the Recurrence free survival (RFS) curve of LN+/LN- and High/Low stromal score samples.

Table S1. The primer sequences of Key genes of StrLNM signature.

Table S2. Stromal score, Immune score, and Estimated score of patients in the TCGA-BLCA cohort.

Table S3. Expression matrix of the training cohort.

Table S4. Expression matrix of the test cohort .

Table S5. The coeﬃcient of each feature in the StrLNM signature

Table S6. Uni-cox and multi-cox analysis of mutation of C3orf70.

Table S7. P value of the correlation between StrLNM key genes and survival
